# Supplementary material for: The social learning and development of intra- and inter-ethnic sharing norms in the Congo Basin: A registered report protocol
Source: PLoS One. 2022 Nov 15;17(11):e0276845. doi: 10.1371/journal.pone.0276845 (PMC9665382; doi:10.1371/journal.pone.0276845)
Supplement: Attachment — Submitted filename: PLOS review.docx [file pone.0276845.s002.docx]

Thank you very much for this thoughtful and encouraging review. In response to your suggestions, we feel we have made several changes which greatly improve the manuscript. Specifically, we have (1) expanded the ethnographic aspect to better capture local norms and spatial and temporal changes in these, and (2) clarified aspects of our experimental and quantitative design, and potential limitations to these. In addition, we have (3) improved the figures and (4) outlined our plans for global inclusivity in research.

We would like to note that one of the co-first authors (Sheina Lew-Levy) has recently changed institutions. Ethical approval for this project will thus need to be sought at the new institution. We will tend to this immediately, and plan to change the ethical approval from Simon Fraser University to Durham University before publication of our registered report (if accepted).

Please find below detailed responses to your queries and suggestions.

Editor Comments

1. Please ensure that your manuscript meets PLOS ONE's style requirements, including those for file naming. The PLOS ONE style templates can be found at
   <https://journals.plos.org/plosone/s/file?id=wjVg/PLOSOne_formatting_sample_main_body.pdf> and 
   <https://journals.plos.org/plosone/s/file?id=ba62/PLOSOne_formatting_sample_title_authors_affiliations.pdf>

We have reviewed these and changed the formatting accordingly.

2. Please include a complete copy of PLOS’ questionnaire on inclusivity in global research in your revised manuscript. Our policy for research in this area aims to improve transparency in the reporting of research performed outside of researchers’ own country or community. The policy applies to researchers who have travelled to a different country to conduct research, research with Indigenous populations or their lands, and research on cultural artefacts. The questionnaire can also be requested at the journal’s discretion for any other submissions, even if these conditions are not met.  Please find more information on the policy and a link to download a blank copy of the questionnaire here: [https://journals.plos.org/plosone/s/best-practices-in-research-reporting](https://journals.plos.org/plosone/s/best-practices-in-research-reporting). Please upload a completed version of your questionnaire as Supporting Information when you resubmit your manuscript.

We have completed the questionnaire and added a section about inclusivity in global research in the main text of the manuscript. We commend PLOS on this initiative.

3. Thank you for stating the following financial disclosure:
    "SLL was funded by a postdoctoral fellowship from the Alexander von Humboldt Foundation. DH was funded by the Max Planck Society for the Advancement of Science."

Please state what role the funders took in the study.  If the funders had no role, please state: "The funders had no role in study design, data collection and analysis, decision to publish, or preparation of the manuscript." 
If this statement is not correct you must amend it as needed. 
Please include this amended Role of Funder statement in your cover letter; we will change the online submission form on your behalf.

The statement is correct. We have added it to the financial disclosure.

4. We note that you have stated that you will provide repository information for your data at acceptance. Should your manuscript be accepted for publication, we will hold it until you provide the relevant accession numbers or DOIs necessary to access your data. If you wish to make changes to your Data Availability statement, please describe these changes in your cover letter and we will update your Data Availability statement to reflect the information you provide.

Please note that there is currently no data, as it has yet to be collected. Our manuscript includes a github repository for the analysis code. We will make all data available in the final registered report.

5. PLOS requires an ORCID iD for the corresponding author in Editorial Manager on papers submitted after December 6th, 2016. Please ensure that you have an ORCID iD and that it is validated in Editorial Manager. To do this, go to ‘Update my Information’ (in the upper left-hand corner of the main menu), and click on the Fetch/Validate link next to the ORCID field. This will take you to the ORCID site and allow you to create a new iD or authenticate a pre-existing iD in Editorial Manager. Please see the following video for instructions on linking an ORCID iD to your Editorial Manager account: <https://www.youtube.com/watch?v=_xcclfuvtxQ>

We have added ORCID for the corresponding author in the editorial manager.

6. Please ensure that you include a title page within your main document. You should list all authors and all affiliations as per our author instructions and clearly indicate the corresponding author

This has been amended.

7. Please review your reference list to ensure that it is complete and correct. If you have cited papers that have been retracted, please include the rationale for doing so in the manuscript text, or remove these references and replace them with relevant current references. Any changes to the reference list should be mentioned in the rebuttal letter that accompanies your revised manuscript. If you need to cite a retracted article, indicate the article’s retracted status in the References list and also include a citation and full reference for the retraction notice.

We have reviewed the reference list.

Reviewer 1

1. In this RR at least, the team appears to be giving far more brain time (if reflected by space used in the RR) to the DG relative to the interview. Given the team makeup, I'm sure they'll make good use of their opportunities in the field, but just in case this slipped under the radar, here's what I'm noticing: The team flagged the comparative dearth of ethnographic data on Bandongo social learning of sharing norms, but hasn't laid out any plans to remedy the situation. This seems important because the DG can only take us so far, telling us the timing of when a specific kind of sharing is acquired -- not trade, for example, and not multi-recipient sharing of parts of a carcass, but just division of a windfall between two people. While the team plans to learn how and from whom intragroup and intergroup sharing is learned, they're leaning hard on quantitative measures that work well with a Fisher's exact test. Ethnographic richness, including free responses from less-shy participants, could add ethnographic richness and help the researchers really understand the nature and life-course of norm acquisition. They could ask questions like:

-- In what context are norms acquired, for example? [Observation can happen in myriad places]
-- What does interethnic sharing mean to you? [...just to examine whether the researcher's interpretation of their observations of interethnic sharing matches what participants think are going on.]
-- Are kids learning differently today than they did in the past? And...
-- Are interethnic interactions between BaYaka and Bandongo *different* today than they were in the past? [Secular trends could be sliding past the researchers that would be informative to any differences between kids and adults.]
...of course this, and what I'm about to suggest below, could be difficult to pull off given the team is relying on research assistants, in which case these may be moot points.

To understand the contexts in which sharing is learned, we have added the following to the quantitative interview (P16):

For both intra-ethnic and inter-ethnic sharing, we will also ask participants to free-list the cultural models (as categories, e.g. mother, friend) from whom they learned to share, and the contexts in which they learned to share [79].

We will analyze that data as follows (P24):

We will report the total number and percent of adult participants who reported learning intra- vs. inter-ethnic sharing in each learning context, separated by participant ethnicity. To investigate variation in learning contexts for intra- vs. inter-ethnic sharing norms, we will conduct 2X2 Fisher’s exact tests on the BaYaka and Bandongo samples separately.

We have now added the following qualitative aspects to the data collection procedure for adults (P16):

Following these structured questions, we will use an open-ended (i.e., ethnographic) format to understand secular changes in inter-ethnic sharing norms across generations, and whether mobility shapes inter-ethnic sharing norms. Questions will include: (1) How did the elders (bakoko) share across ethnicity? (2) How do you share across ethnicity? (3) Are children learning to share differently than in the past? (4) How did BaYaka and Bandongo interact in the past? (5) How have these interactions changed? (6) How do you resolve a conflict related to inter-ethnic sharing today? (7) How were these conflicts resolved in the past? (8) Do BaYaka/Bandongo who live in the forest full-time or almost full-time have different intra- and inter-ethnic sharing norms than those who spend more time in the village?

We will analyze these qualitative data as follows (P23):

Answers to the open-ended (i.e., ethnographic) questions posed to adults and children regarding learning, cooperation, mobility, and inter-generation changes will be transcribed. Salient qualitative trends will be identified and summarized.

2. Relatedly, assuming child participants aren't *too* shy and just really want to get out of the research house after the DG wraps up, the researchers could think more about how to get broadly useful data from the post-game interview. For example, other yes/no questions the children could answer that could again add to ethnographic richness are things like:
   -- Did you learn interethnic sharing from your mom?
   -- Does interethnic sharing mean X to you?
   ...things that (in addition to their ethnographic observations) allow the team to check whether kids are learning in the same way as adults report *they* learned in the past in the interview -- again, thinking about secular trends and changes in intergroup interaction over time.

In line with points #1 and #2, we have also added the following to the qualitative aspects to the data collection procedure for children (P17):

In addition, we will ask child participants open-ended (i.e., ethnographic) questions including: (1) From whom did you learn intra-ethnic sharing norms? (2) From whom did you learn inter-ethnic sharing norms? (3) How do you share within ethnicity? (4) How do you share across ethnicity? While we expect fewer children will answer these questions due to shyness, a small sample of answers will nonetheless ensure that we are able to track secular trends regarding changes in the social learning of sharing norms over time.

3. This is a tall ask, and I don't expect the team to necessarily address it, but at least to think about it if they haven't already. The team is attending to things like participant shyness and mobility that might affect sample size. What about self-selection in this regard -- is there any reason to think that candidate participants who acquired interethnic norms earlier, through a different means, or (per my suggestions above) even have a different *set* of interethnic norms would be more mobile or less shy? This is what I would expect a priori, and I wonder if the researchers have any insight into whether self-selection on these lines (or others) would affect their results.

Thanks for this question. We’ve clarified our view on this matter as follows (P14):

Note that inter-ethnic cooperation generally, and sharing specifically, is highly regulated, easily observable in daily interactions, and widely practiced across a range of forest- and village-based contexts and resources. Thus, we do not a priori expect participant mobility and shyness to co-vary with inter-ethnic sharing norms, nor how these norms are acquired. Local perspectives on this potential limitation will nonetheless be elicited during the ethnographic interviews (see below).

We have also added this to the ethnographic interview. Please see question 8 in response to review point 1.

4. Sample size will be sufficient if a few conditions hold: the transition from infant to adult state isn't very fast and/or the difference between intergroup and intragroup norm acquisition is four years. Four years is a while developmentally. Do the researchers have any thoughts on how to disambiguate between a quick transition vs similarity in timing of acquisition if results are inconclusive? For example, per the above, it seems that ethnography could really be an asset in that circumstance, including adult's interview responses about when they learned the relevant norms.

Thanks for this insight. We’ve now clarified our thinking on this matter in the text as follows (P29):

In the absence of relevant previous studies, it is difficult to assess in advance how likely our planned sample size is to yield a confident detection of difference in norm completion ages. Still, the above analysis shows such a detection is certainly not impossible in principle. Note that even if we are unable to confidently identify a difference in norm completion ages from these data, other differences in the developmental trajectory may still be identifiable. Further, the fitted model will provide estimates with quantified uncertainty of all aspects of the developmental process for both societies, including the various sharing rates. These estimates are still potentially ethnographically informative, and can be combined or compared with the results of ethnographic interviews. In particular, if the model is able to make confident statements about some aspects of norm acquisition which corroborate the accounts given in interviews, then those interviews may help to disambiguate other aspects where the model is less confident.

5. Just to flag this: the team mentions ethnic markers at the outset and never addresses how markers come into play (or not) in BaYaka and Bandongo interactions, but implicitly leans on ethnic markers in the game design, treating red as one ethnic group and blue as another. Presumably markers are salient to BaYaka and Bandongo participants, as they seem to be (in some form or another) to all humans, but establishing this might check the ecological validity of the game (although I'm fine with the assumption that humans everywhere can work with this -- again, just flagging the assumption).

We now clarify this in the ethnographic section as follows (P8):

BaYaka and Bandongo primarily use linguistic (Yaka and Bondongo/Lingala) and behavioural ethnic markers to distinguish between their communities. BaYaka view Bandongo as accumulators of wealth, hierarchical, and as claimants of forest areas as their own [75]. Bandongo identify BaYaka based on their sociability, lack of food reserves (reflecting their immediate-return economy), and their knowledge of the forest [75].

We now also justify the use of randomly assigned colours in the methods (P18-19):

We have opted to use these randomly-assigned visual markers of ethnicity because, as previously mentioned, BaYaka and Bandongo primarily distinguish each other through linguistic and behavioural ethnic markers, which do not straightforwardly translate to this experimental paradigm. Randomly-assigned colours have been used successfully as in- and out-group markers in previous experiments in the post-industrialized west [51], and are also used as part of team uniforms at the field site during inter-village football games. Thus, we expect that assigning meeple colours to ethnicity will be well understood in this context.

6. At one point, the authors end a paragraph about intergroup interactions in children by saying "Inter-group cooperation may be enhanced in communities where strong social norms regulate inter-ethnic interactions" but don't provide citations. When the time comes, they can use two they already have -- Fearon & Laitin, and Bunce (although the 2018 Bunce paper may be better) -- and can also consider any paper about how religion sets norms for intergroup interactions (e.g., Islam in East Africa is a classic -- see Ensminger 1992 Making a Market) and some classic stuff by Barth (e.g., Barth 1956 Ecologic Relationships of Ethnic Groups in Swat, North Pakistan).

Thanks! We now cite Bunce 2018 here.

7. Something to consider: the lag time between research assistant training and project commencement is a bit long (six months-ish). I get the sense that the team does this all the time, so I'm sure they know the best way to go about this, but a refresher for the RAs could be useful before things get started in early 2023.

We’ve now added reference to a refresher (P30):

We will train research assistants in July-August 2023. After a refresher in experimental methods, data will be collected in early 2024, when most villagers have returned from their fishing camps, and thus, the population of each village is at its highest. Data will be analyzed in March-April 2024. We aim to submit the final manuscript with data and analyses in September 2024. Our timeline will adjust to European and Congolese COVID-related regulations regarding safe travel.
